# Case Report and literature review: immune checkpoint inhibitor-associated myasthenia gravis and myocarditis

**DOI:** 10.3389/fcvm.2026.1764567

**Published:** 2026-03-19

**Authors:** Zhiyue Shang, Shuai Hao, Jianhong Wang, Weirong Li

**Affiliations:** 1 Department of Neurology, Cardiovascular Hospital Affiliated to Shanxi Medical University; Shanxi Key Laboratory of Heart Failure Precision Medicine, Taiyuan, Shanxi, China; 2 Department of Pharmacy, Cardiovascular Hospital Affiliated to Shanxi Medical University; Shanxi Key Laboratory of Heart Failure Precision Medicine, Taiyuan, Shanxi, China

**Keywords:** adenocarcinoma, case report, immune checkpoint inhibitor, immune-related adverseevents, myasthenia gravis, myocarditis

## Abstract

To investigate the clinical features of immune checkpoint inhibitor (ICI)-associated myasthenia gravis (MG) with concurrent myocarditis with the aim of enhancing our understanding. We retrospectively analyzed the clinical data of a patient diagnosed with ICI-associated MG and myocarditis, and reviewed the relevant literature. A 67-year-old man was admitted with a 4-day history of right-sided ptosis and blurred vision. His medical history included resection of adenocarcinoma in the right upper lung lobe over 18 years ago and a diagnosis of new lung cancer in the right lower lobe four months prior to presentation. In May 2025, lung cancer recurrence was identified but the patient declined any treatment at that time. In September 2025, following radiotherapy and a single dose of serplulimab (a programmed cell death protein 1 inhibitor, PD-1), the patient developed clinical symptoms including ptosis, a positive fatigue test, and a positive neostigmine test, suggestive of myasthenia gravis. Biochemical tests, electrocardiogram (ECG), and echocardiography indicated myocarditis. The clinical diagnosis was ICI-associated myocarditis. Management involved the discontinuation of immunotherapy, administration of glucocorticoids, and symptomatic supportive treatment, which led to clinical improvement. A literature review summarized 45 cases, indicating that this overlapping immune-related adverse event is rare and has a poor prognosis. Multiple adverse reactions to ICIs can occur simultaneously. Prompt recognition of symptoms and initiation of targeted and effective treatments are crucial for optimizing clinical outcomes.

## 1 Introduction

Immune checkpoint inhibitors (ICIs) include monoclonal antibodies targeting programmed death-1 (PD-1), programmed death-ligand 1 (PD-L1), and cytotoxic T-lymphocyte-associated protein-4 (CTLA-4), among others (1). Classic immune checkpoint molecules, such as PD-1 and CTLA-4, play a crucial role in maintaining immune homeostasis by delivering inhibitory signals to T cells, which prevents their overactivation (2). However, tumor cells exploit this mechanism by producing high levels of these immune checkpoint ligands. This suppresses the activity of cytotoxic *T* cells, thus allowing immune evasion. Therefore, blocking the transmission of these checkpoint signals can enhance the immune system's attack on tumors and has become a pivotal treatment strategy (3). Since the first immune checkpoint inhibitor, ipilimumab, was approved by the U.S. FDA for the treatment of melanoma in May 2011, significant progress has been made in the development of ICIs. Several agents have subsequently entered the market, including pembrolizumab, nivolumab, atezolizumab, durvalumab, and avelumab. Among these, nivolumab and pembrolizumab were approved in China in May and July 2018, respectively, and have demonstrated remarkable efficacy in cancers such as melanoma, lung cancer, and renal cell carcinoma (4–9).

ICIs have become an important class of cancer therapies due to their demonstrated efficacy. However, the mechanism that activates immune cells against tumors can also lead to excessive immune responses. This overactivation may cause autoimmune injury to multiple organ systems, a group of side effects referred to as immune-related adverse events (irAEs) (10).The precise pathophysiological mechanisms underlying irAEs are not yet fully understood (11). The development of irAEs likely involves multiple mechanisms. These include the activation of cytotoxic T lymphocytes against normal tissues and the activation of autoreactive B lymphocytes, leading to the production of autoantibodies that mediate damage, potentially via the complement cascade (12). Other contributing factors include molecular mimicry (where ICIs bind to off-target sites such as CTLA-4 on healthy cells) and dysregulated production of cytokines that trigger pro-inflammatory pathways (e.g., JAK-STAT and PI3K-AKT-mTOR) (13). Furthermore, factors such as intestinal dysbiosis and microbiota-derived metabolites may contribute to aberrant immune activation, predisposing patients to irAEs during ICI therapy (14).

In fact, irAEs can affect virtually any organ system. The gastrointestinal tract and skin are among the most frequently involved sites. Other potential manifestations include rheumatic/musculoskeletal toxicities, hypophysitis, myocarditis, pneumonitis, hepatitis, and adrenalitis, among others. Although cardiac complications represent a very rare form of irAE, they are associated with a relatively high case fatality rate, ranging from 27% to 60%, compared to other irAEs (15). ICIs can also induce neurological immune-related diseases, such as autoimmune encephalitis, Guillain-Barré syndrome, peripheral sensorimotor neuropathy, posterior reversible encephalopathy syndrome, aseptic meningitis, and transverse myelitis, among others (16). Myasthenia gravis (MG) is a form of neurological irAEs, but its incidence is much lower than that of irAEs affecting other organs. However, ICI-associated MG carries a high mortality rate, primarily due to its rapid progression. Therefore, early diagnosis and treatment are critical (17).

## 2 Case description

A 67-year-old man was admitted to the Department of Neurology at Shanxi Cardiovascular Hospital on September 26, 2025, presenting with a 4-day history of right-sided ptosis and blurred vision (Table 1).

**Table 1:** Timeline of care of the patient.

Date	Event
2007-11	Right upper lobectomy performed for lung adenocarcinoma; followed by one year of immunomodulatory therapy postoperatively.
2008-11, 2009-04, 2009-08, 2009-12, 2010-07, 2011-07, 2012-09-14, 2013-11-12	Follow-up examination showed no signs of obvious recurrence or metastasis.
2020	Diagnosed with coronary heart disease; underwent PCI with stent implantation.
2025-05	PET-CT scan suggested recurrence of lung cancer; the patient declined further treatment.
2025-06-26	Bronchoscopy performed; pathology indicated lung adenocarcinoma; immunohistochemistry and genetic testing revealed KRAS G12C mutation.
2025-07-28	Treatment regimen adjusted to Serplulimab 300 mg once + Pemetrexed 0.8 g once + Nedaplatin 30 mg qd (from July 31 to August 3, 2025) for one cycle, along with intensity-modulated radiotherapy to the right lung lesion.
2025-09-22	Symptom onset: Right-sided ptosis and blurred vision.
2025-09-26 (Day 1)	Hospital admission. Investigations performed: ECG, cranial MRI, echocardiography, and RNS.
2025-09-27 (Day 2)	ECG and cardiac biomarker tests.
2025-09-28 (Day 3)	Cardiac biomarker tests. Treatment initiated: Intravenous methylprednisolone sodium succinate (120 mg daily).
2025-09-30 (Day 5)	Cardiac biomarker tests.
2025-10-01 (Day 6)	Intravenous methylprednisolone sodium succinate discontinued. Treatment adjusted: Switched to oral prednisone acetate (60 mg daily).
2025-10-07 (Day 12)	Cardiac biomarker tests. Treatment adjusted: Oral prednisone acetate dose reduced to 55 mg daily.
2025-10-13 (Day 18)	Cardiac biomarker tests and 18-lead ECG performed. Treatment adjusted: Oral prednisone acetate dose reduced to 50 mg daily.
2025-10-21 (Day 26)	Cardiac biomarker tests. Treatment adjusted: Oral prednisone acetate dose reduced to 45 mg daily.
2025-10-28 (Day 33)	Cardiac biomarker tests. Follow-up: No recurrence of myocarditis or MG was observed.

The patient had a 30-pack-year smoking history (averaging 20 cigarettes per day) and had quit smoking 18 years prior. His family history was notable for a deceased brother with lung cancer. There was no family history of genetic, autoimmune, or psychiatric diseases.

The patient was diagnosed with adenocarcinoma of the right upper lung lobe and underwent a right upper lobectomy 18 years prior. Postoperative pathological examination confirmed nodular adenocarcinoma of the right upper lobe, with no carcinoma involvement at the bronchial stump or in the examined lymph nodes. He recovered well postoperatively and was then transferred to the Department of Biotherapy at Shanxi Cancer Hospital for immunomodulatory therapy, which consisted of subcutaneous injections of thymosin alpha-1 (1.6 mg, twice weekly) for approximately one year. He underwent multiple follow-up visits at the same department on the following dates: November 2008, April 2009, August 2009, December 2009, July 2010, July 2011, September 14, 2012, and November 12, 2013. No signs of obvious recurrence or metastasis were observed during these visits, and subsequent regular follow-up examinations revealed no recurrence or metastasis.

He had a history of coronary heart disease diagnosed in 2020, for which he underwent percutaneous coronary intervention (PCI) during the same period. He had been on long-term regular medication with aspirin enteric-coated tablet (100 mg once daily in the morning) and rosuvastatin calcium tablet (10 mg once daily in the evening).

**Figure 1:**
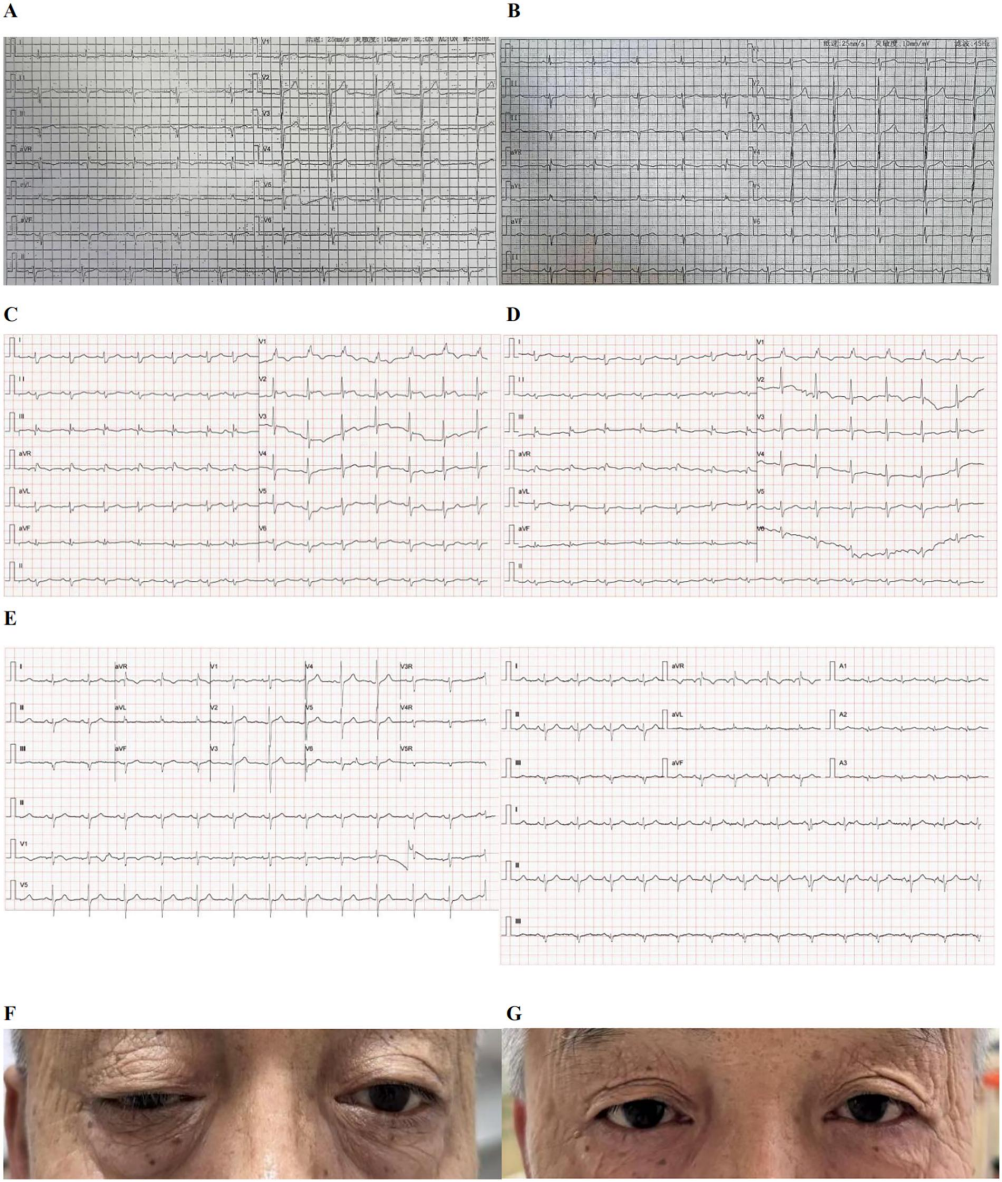
Evolution of ECG and right eyelid ptosis before and after treatment. **(A)** ECG on May 30, 2025 (Shanxi Cancer Hospital): Essentially normal tracing showing sinus rhythm, left axis deviation, and occasional atrial premature beats. **(B)** ECG on July 28, 2025 (Shanxi Cancer Hospital): Essentially normal tracing showing sinus rhythm and left axis deviation. **(C)** ECG on admission day (September 26, 2025, Shanxi Cardiovascular Hospital): Essentially normal tracing showing sinus rhythm, right axis deviation, and incomplete right bundle branch block. **(D)** ECG on the following day (September 27, 2025, Shanxi Cardiovascular Hospital): Abnormal tracing showing sinus rhythm, right axis deviation, complete right bundle branch block, and ST-T changes. **(E)** ECG at follow-up (Day 18 post-treatment, 18-lead ECG, Shanxi Cardiovascular Hospital): Essentially normal tracing showing sinus rhythm and left axis deviation. **(F)** Noticeable right-sided ptosis is observed prior to treatment. **(G)** The ptosis has significantly resolved following immunosuppressive therapy.

In May 2025, a positron emission tomography-computed tomography (PET-CT) scan was performed at the outpatient clinic of Shanxi Cancer Hospital due to shortness of breath. It revealed an abnormal density (2.6 × 2.9 × 3.7 cm) with uniformly increased metabolism at the opening of the right lower lobe bronchus, surrounded by scattered nodules showing mildly increased metabolism. These findings were suggestive of recurrent lung cancer. Electrocardiogram (ECG) showed essentially normal findings (Figure 1A). Echocardiography revealed segmental wall motion abnormalities, characterized by increased echogenicity and hypokinesia of the inferoposterior wall of the left ventricle. Left ventricular systolic function was at the lower limit of normal, with an ejection fraction (EF) of 51.71%. Laboratory tests revealed the following cardiac biomarker levels: cardiac troponin I (cTnI), 0.11 ng/mL (reference range: 0.00–0.30 ng/mL); myoglobin, 20.08 ng/mL (reference range: 0.00–58.00 ng/mL); and creatine kinase-MB (CK-MB), 1.59 ng/mL (reference range: 0.00–5.00 ng/mL). The level of N-terminal pro-B-type natriuretic peptide (NT-proBNP) was 85 ng/L (reference range: 0–125 ng/L). The patient declined further treatment at that time and was discharged.

On June 26, 2025, the patient underwent bronchoscopy at Beijing Cancer Hospital, which revealed an irregular, heterogeneous, hypoechoic lesion (max cross-sectional diameter >1.5 × 1.2 cm with internal punctate vascularity) external to the airway lumen of the right lower lobe superior segment. Pathological and immunohistochemical (IHC) analyses of the biopsy supported a diagnosis of lung adenocarcinoma, showing the following profile: *ALK* (Ventana) negative [positive control (+), negative control (−)], *BRAF* negative, *c-MET* (2+), *HER2* (1+) [positive control (3+), negative control (0)], PD-L1 (22C3) with a Tumor Proportion Score (TPS) of 5% [positive control (+), negative control (−)], ROS-1 negative, Ki-67 (+10%), and TTF-1 positive. Subsequent genetic testing by ARMS-PCR method identified a *KRAS* G12C mutation (Exon 2) while revealing no detected mutations in *EGFR*, *BRAF*, *NRAS*, *PIK3CA*, or *HER2*, and no detected *ALK*, *ROS1*, or *RET* fusions or *MET* exon 14 skipping mutation.

He was readmitted to Shanxi Cancer Hospitalon on July 28, 2025. ECG showed essentially normal findings (Figure 1B). Systemic therapy was initiated on July 31, 2025, with one cycle of serplulimab (300 mg once only), pemetrexed (0.8 g once only), and nedaplatin (30 mg once daily from July 31 to August 3, 2025). Subsequently, from August 19 to September 25, 2025, the patient underwent intensity-modulated radiotherapy (IMRT) targeting the right lung lesion. The prescribed doses were: 95% of the planning target volume (PTV) received 50.4 Gy in 28 fractions of 1.8 Gy, and 95% of the Planning Gross Tumor Volume (PGTV) received 59.92 Gy in 28 fractions of 2.14 Gy.

On September 26, 2025, the patient's vital signs were as follows: body temperature 36.3°C, pulse 80 beats per minute, respiratory rate 18 breaths per minute, and blood pressure 120/80 mmHg. His height was 168 cm and weight was 67.5 kg. Cardiopulmonary and abdominal examinations were unremarkable. No edema was present in the lower extremities.

Neurological examination showed the patient was alert, oriented, and had normal speech. Right-sided ptosis, covering the cornea from the 9 to 3 o'clock positions, was noted and worsened with fatigue. Both pupils were equal, round, and reactive to light. Forehead wrinkles and nasolabial folds were symmetrical bilaterally. The tongue was midline. Diaphragmatic strength was intact with a strong cough. Muscle strength was grade 5 in all extremities, with normal tone and coordination. Sensation was intact symmetrically. Deep tendon reflexes were symmetric, and no pathological reflexes were elicited. The fatigue test was positive.

On admission, ECG showed sinus rhythm with incomplete right bundle branch block (Figure 1C). Echocardiography revealed a left ventricular ejection fraction (LVEF) of 51% with akinesis of the basal and mid segments of the inferoposterior and lateral walls. Cardiac magnetic resonance (CMR) was not performed due to limited availability at the institution. Cranial magnetic resonance imaging (MRI) revealed lacunar infarcts in the periventricular white matter and the left frontal subcortical region, localized stenosis of the right internal carotid artery siphon, and intermittent visualization of the left vertebral artery. Chest CT findings were consistent with post-lobectomy changes, presenting as bands in the lingular segment of the left upper lobe and the basal segments of the right lower lobe, subpleural interstitial changes in the basal segments of both lower lobes, and a small solid nodule (approximately 8 mm × 5 mm) in the superior lingular segment of the left upper lobe. Repetitive Nerve Stimulation (RNS) results were normal. Both the neostigmine test and the eyelid fatigue test were positive.

On September 27, 2025, ECG showed sinus rhythm, right axis deviation, complete right bundle branch block, and ST-T changes (Figure 1D). Laboratory tests revealed markedly elevated cardiac biomarkers: high-sensitivity cardiac troponin (hs-cTn), 736.12 ng/L (reference range: 0–42.9 ng/L); myoglobin (MYO), 1255.3 μg/L (reference range: 17.4–105.7 μg/L); creatine kinase (CK), 2286.0 U/L (reference range: 50.0–310.0 U/L); CK-MB, 73.1 U/L (reference range: 0.0–25.0 U/L); α-hydroxybutyrate dehydrogenase (*α*-HBDH), 395.8 U/L (reference range: 90.0–197.0 U/L); and lactate dehydrogenase (LDH), 444.9 U/L (reference range: 120.0–250.0 U/L). NT-proBNP level was 75 ng/L (reference range: 0–125 ng/L). Additional results were as follows: complete blood count: leukocyte count (WBC), 5.75 × 10^9^/L (reference range: 3.50–9.50 × 10^9^/L); erythrocyte count (RBC), 4.51 × 10^12^/L (reference range: 4.30–5.80 × 10^12^/L); hemoglobin (HGB), 147 g/L (reference range: 130–175 g/L); platelet count (PLT), 173 × 10^9^/L (reference range: 125–350 × 10^9^/L); neutrophil percentage (NEUT%), 78.5% (reference range: 40.0%–75.0%); lymphocyte percentage (LYMPH%), 7.5% (reference range: 20.0%–50.0%); urinalysis: normal; lipid profile: total cholesterol (TC), 4.29 mmol/L (reference range: <5.20 mmol/L); triglycerides (TG), 2.14 mmol/L (reference range: <1.70 mmol/L); high-density lipoprotein cholesterol (HDL-C), 1.00 mmol/L (reference range: ≥1.04 mmol/L); low-density lipoprotein cholesterol (LDL-C), 2.69 mmol/L (reference range: <1.4 mmol/L for very high-risk, <1.8 mmol/L for high-risk, <2.6 mmol/L for moderate-risk, <3.4 mmol/L for low-risk individuals); other serum parameters: alanine aminotransferase (ALT), 62.9 U/L (reference range: 9.0–50.0 U/L); creatinine (Cr), 71.2 μmol/L (reference range: 57.0–111.0 μmol/L); uric acid (UA), 203.0 μmol/L (reference range: 208.0–428.0 μmol/L); potassium (K), 4.45 mmol/L (reference range: 3.50–5.30 mmol/L); sodium (Na), 145.0 mmol/L (reference range: 137.0–147.0 mmol/L); homocysteine (HCY), 15.4 μmol/L (reference range: 0.0–15.0 μmol/L); coagulation studies: normal [D-dimer (DD2): 490.0 μg/L (reference range: 0.0–500.0 μg/L)]; vitamin B12 (B12), 220 pmol/L (reference range: 133–675 pmol/L).

## 3 Clinical management

### 3.1 Diagnostic approach

Given the patient's concurrent cardiovascular and neurological symptoms following ICI therapy, a systematic work-up was initiated to evaluate for possible irAEs. The diagnostic criteria for ICI-associated myocarditis and MG were applied as follows.

The diagnosis of ICI-associated myocarditis was based on established criteria (18). The pathological gold standard is endomyocardial biopsy (EMB), defined histologically by multifocal inflammatory cell infiltrates (exceeding 14 cells per high-power field) with accompanying cardiomyocyte necrosis. For clinical diagnosis, an elevated serum cardiac troponin level (a new or significant increase from baseline) is required, plus either one major criterion or at least two minor criteria, after excluding acute coronary syndrome and acute infectious causes. The major criterion is cardiac magnetic resonance (CMR) findings consistent with acute myocarditis per the modified Lake Louise Criteria. Minor criteria include: (a) a compatible clinical syndrome (e.g., fatigue, chest pain, dyspnea, palpitations, syncope); (b) new-onset ventricular arrhythmias or conduction system disease; (c) a decline in systolic function without a Takotsubo pattern; and (d) concurrent immune-related adverse events, particularly myositis, myopathy, or MG. A definitive clinical diagnosis can be established when troponin elevation coexists with CMR-confirmed inflammation or a combination of clinical and paraclinical minor criteria.

The diagnosis of MG was evaluated according to established criteria (19). Diagnosis is confirmed when a patient exhibits one or more characteristic clinical symptoms, specifically fluctuating skeletal muscle weakness and fatigability (Criterion A), and fulfills at least one objective criterion from either pathogenic autoantibodies (Criterion B) or impaired neuromuscular transmission (Criterion C). Criterion B requires seropositivity for anti-acetylcholine receptor (AChR) or anti-muscle-specific tyrosine kinase (MuSK) antibodies. Criterion C can be met by a positive neostigmine test, abnormal repetitive nerve stimulation (RNS), or increased jitter on single-fiber electromyography (SFEMG). A positive response to immunotherapy serves as supportive evidence (Criterion D).

Based on the clinical presentation and investigative findings, the patient was diagnosed with concurrent immune-related adverse events. ICI-associated myocarditis was diagnosed following the observation of new-onset incomplete right bundle branch block and elevated high-sensitivity cardiac troponin, and after the exclusion of acute myocardial infarction and acute infectious myocarditis through ECG, echocardiography, and cardiology consultation. The clinical presentation of ptosis further supported the diagnosis of an irAE. MG was diagnosed by neurologists based on characteristic right-sided ptosis, the temporal association with ICI therapy, and positive results on specific tests (RNS, neostigmine test, eyelid fatigue test). Despite elevated muscle enzymes, ICI-related myositis was ruled out due to the absence of myalgia or limb weakness.

### 3.2 Therapeutic management

The patient was diagnosed with ICI-associated MG and myocarditis. Serplulimab immunotherapy was immediately discontinued. On September 28, 2025, intravenous methylprednisolone sodium succinate was initiated at a dose of 120 mg daily for three days, followed by conversion to oral prednisone acetate at 60 mg/day. The oral corticosteroid dose was subsequently tapered by 5 mg per week until discontinuation. Adjunctive therapies, including gastric acid suppression and potassium and calcium supplementation, were administered to manage potential glucocorticoid-related side effects. Following the discontinuation of ICI therapy, the patient received no further antitumor therapy.

### 3.3 Treatment response and follow-up

During hospitalization, treatment adherence was ensured by direct administration; for the outpatient corticosteroid taper, tolerability and adherence were monitored through clinical follow-up and serial biomarker testing. Following therapy initiation, the patient's elevated cardiac enzymes, biomarkers, and ECG findings progressively normalized (Table 2; Figure 1E). Concurrently, the symptoms of right-sided ptosis significantly improved and eventually resolved completely (Figures 1F,G). The patient expressed relief and gratitude upon discharge, as his right-sided ptosis and blurred vision had completely resolved, reflecting that he had mistakenly attributed his initial symptoms to cancer progression rather than to treatment-related immune-related complication.

**Table 2:** Serial measurements of cardiac enzymes and biomarkers during the clinical course.

Timepoint	2025-09-27 (Day 2)	2025-09-28 (Day 3)	2025-09-30 (Day 5)	2025-10-07 (Day 12)	2025-10-13 (Day 18)	2025-10-21 (Day 26)	2025-10-28 (Day 33)
hs-cTnI (ng/L)	736.12	694.09	191.36	43.49	25.36	18.16	17.59
MYO (μg/L)	1255.3	-	518	-	147.2	-	-
CK (U/L)	2286	806.0	-	-	219.0	-	82.0
α-HBDH (U/L)	395.8	399.0	-	-	205.2	-	-
LDH (U/L)	444.9	419.0	-	-	321.8	-	-
CK-MB (U/L)	73.1	40.0	-	-	63.5	-	29.8

hs-cTnI, high-sensitivity cardiac troponin I; MYO, myoglobin; CK, creatine kinase; α-HBDH, alpha-hydroxybutyrate dehydrogenase; LDH, lactate dehydrogenase; CK-MB, creatine kinase-MB isoenzyme; IV, intravenous; MP, methylprednisolone; -, not tested.

Reference ranges are as follows: hs-cTnI, 0–42.9 ng/L; Myo, 17.4–105.7 μg/L; CK, 50.0–310.0 U/L; α-HBDH, 90.0–197.0 U/L; LDH, 120.0–250.0 U/L; CK-MB, 0.0–25.0 U/L.

## 4 Literature review

A literature search was conducted using the PubMed database for articles published between 2015 and 2025 using the keywords “immune checkpoint inhibitors,” “myocarditis,” and “myasthenia gravis.” Among the retrieved publications, studies were excluded if they did not clearly document a concurrent diagnosis of ICI-associated MG and myocarditis in the same patient. Additional exclusion criteria were a lack of detailed information on patient demographics, underlying malignancy, types of ICIs administered, treatment regimens, or clinical outcomes. Eligible cases were analyzed to summarize their clinical characteristics, types of monoclonal antibodies involved, treatment strategies, and prognosis.

The clinical characteristics of the patients are summarized in Table 3. A total of 45 cases have been reported in the literature. The cohort comprised 30 males and 15 females, with an age range of 30–89 years and a mean age of 68 years. The most common primary malignancies were melanoma (*n* = 17), thymoma (*n* = 5), and non-small cell lung cancer (*n* = 4). Nivolumab (*n* = 19), pembrolizumab (*n* = 11), ipilimumab (*n* = 5), and sintilimab (*n* = 5) were the most frequently administered ICIs.

**Table 3:** Clinical characteristics of reported cases of immune checkpoint inhibitor-associated myasthenia gravis and myocarditis.

Case no. (Reference)	Age (years)	Sex	Cancer type	ICI agent(s)	Treatment(s)	Outcome
1 (53)	80	F	Bladder Cancer	Nivolumab	GC, IVIG, PLEX	Death
2 (54)	78	M	Non-Small Cell Lung Cancer	Durvalumab	GC, IVIG, PLEX	Improved
3 (55)	83	M	Liver Cancer	Atezolizumab	GC, IVIG	Symptom Relief
4 (56)	73	M	Metastatic Melanoma	Nivolumab + Ipilimumab	GC, IVIG, PLEX, Rituximab	Symptom Relief
5 (57)	33	M	Thymoma	Nivolumab	GC, IVIG, AZA	Symptom Relief
6 (58)	51	M	Lung Cancer	Sintilimab	GC, IVIG	Improved
7 (59)	75	M	Lung Adenoma	Pembrolizumab	GC, IVIG, PLEX, Infliximab	Death
8 (60)	71	F	Cholangiocarcinoma	Sintilimab	GC, IVIG	Symptom Relief
9 (61)	65	M	Colon Cancer	Tislelizumab	GC, IVIG	Symptom Relief
10 (62)	81	M	Bladder Cancer	Pembrolizumab	GC, IVIG	Symptom Relief
11 (63)	77	M	Non-Small Cell Lung Cancer	Spartalizumab	GC, IVIG, PLEX	Death
12 (63)	78	M	Prostate Cancer	Pembrolizumab	GC, IVIG, PLEX, Rituximab	Death
13 (63)	70	M	Melanoma	Nivolumab	GC, IVIG, PLEX, CTX	Death
14 (64)	55	F	Melanoma	Nivolumab	GC, IVIG, PLEX	Symptom Relief
15 (65)	65	F	Melanoma	Nivolumab/Relatlimab	GC, PLEX, Abatacept	Symptom Relief
16 (65)	84	M	Melanoma	Nivolumab + Ipilimumab	GC	Death
17 (65)	70	M	Melanoma	Nivolumab/Relatlimab	GC, IVIG, PLEX, Abatacept	Symptom Relief (Died from complications)
18 (66)	71	M	Melanoma	Pembrolizumab	GC, IVIG	Symptom Relief
19 (66)	65	F	Squamous Cell Carcinoma	Pembrolizumab	GC, IVIG	Symptom Relief
20 (67)	69	F	Esophageal Squamous Cell Carcinoma	Camrelizumab	GC, IVIG	Symptom Relief
21 (68)	47	F	Thymoma	Toripalimab	GC	Symptom Relief
22 (69)	77	M	Chordoma	Sintilimab + Anlotinib	GC	Symptom Relief (Died from secondary infection)
23 (69)	69	F	Non-Small Cell Lung Cancer	Bevacizumab + Camrelizumab	GC, IVIG	Death
24 (70)	33	M	Thymoma	Sintilimab	GC, IVIG	Symptom Relief
25 (71)	68	M	Melanoma	Nivolumab	GC, IVIG	Death
26 (72)	72	M	Non-Small Cell Lung Cancer	Durvalumab	GC, PLEX	Symptom Relief
27 (73)	53	F	Melanoma	Ipilimumab	GC, PLEX	Symptom Relief (Died from primary disease)
28 (74)	82	M	Melanoma	Nivolumab	GC, IVIG	Death
29 (75)	67	F	Melanoma	Ipilimumab + Nivolumab	GC, PLEX	Death
30 (75)	89	M	Non-Small Cell Lung Cancer	Pembrolizumab	GC	Symptom Relief
31 (75)	70	M	Melanoma	Ipilimumab + Nivolumab	GC, PLEX, MMF, CTX	Death
32 (75)	70	M	Renal Cell Carcinoma	Ipilimumab + Nivolumab	GC, PLEX	Death
33 (75)	61	F	Breast Cancer	Durvalumab + Tremelimumab	GC, MMF	Death
34 (75)	83	M	Melanoma	Nivolumab	GC	Death
35 (75)	79	M	Melanoma	Pembrolizumab	GC, MMF, IVIG	Death
36 (76)	66	M	Lung Adenocarcinoma	Sintilimab	GC, PLEX	Symptom Relief
37 (77)	86	M	Squamous Cell Carcinoma	Cemiplimab	GC, IVIG	Death
38 (78)	83	M	Melanoma	Pembrolizumab	GC, PLEX	Symptom Relief
39 (79)	72	F	Renal Cell Carcinoma	Nivolumab	GC, IVIG	Death
40 (79)	71	M	Renal Cell Carcinoma	Nivolumab	GC, IVIG	Death
41 (80)	78	NK	Melanoma	Ipilimumab + Nivolumab	GC, IVIG, PLEX	Death
42 (81)	58	F	Thymoma	Pembrolizumab	GC	Death
43 (81)	30	F	Thymoma	Pembrolizumab	GC, IVIG, Rituximab	Symptom Relief (Died from secondary infection)
44 (82)	63	M	Bladder Cancer	Pembrolizumab	GC	Symptom Relief
45 (83)	80	M	Melanoma	Nivolumab	GC, IVIG, PLEX	Symptom Relief

AZA, azathioprine; CTX, cyclophosphamide; F, female; GC, glucocorticoid; ICI, immune checkpoint inhibitor; IVIG, intravenous immunoglobulin; M, male; MMF, mycophenolate mofetil; NK, not known; PLEX, plasma exchange.

All patients received glucocorticoids. A considerable proportion of patients received additional immunosuppressive therapies, primarily intravenous immunoglobulin (*n* = 29) and plasma exchange (*n* = 19). A small subset of patients received three or even four different immunomodulatory agents.

The overall prognosis of patients with these overlapping immune-related complications is poor. Among the 45 summarized cases, 20 (44%) showed a poor response to therapy and died shortly after symptom onset. Among the 25 patients whose immune-related adverse event (irAE) symptoms improved with treatment, a significant proportion (12%, 3/25) died owing to complications such as infection.

## 5 Discussion and conclusion

The rapid increase in the clinical application of ICIs has been accompanied by a significant increase in the reports of irAEs. Common irAEs include dermatological (e.g., dermatitis), gastrointestinal (e.g., colitis), endocrine (e.g., thyroiditis), and respiratory (e.g., pneumonia) toxicities. In contrast, cardiovascular (e.g., myocarditis) and neurological (e.g., MG) irAEs are relatively rare or uncommon (20, 21). The reported incidence of ICI-associated myocarditis ranges from 0.06% to 3.30% (22), while that of ICI-associated MG is approximately 0.24% (23). Although most cases of ICI-associated myocarditis or MG occur independently, a considerable number of patients present with an overlap syndrome. Elevated creatine kinase levels are observed in 36.4% to 90.0% of patients with ICI-associated MG, while concurrent myositis and/or myocarditis occurs in 10.0% to 43.6% of such patients (23–29).

Clinical manifestations of ICI-associated myocarditis vary widely. More than 40% of patients are asymptomatic, whereas 7.4% present with cardiac arrest. Other symptomatic presentations often involve nonspecific symptoms such as palpitations, shortness of breath, chest pain or tightness, and dyspnea (30, 31). The elevation of any myocardial injury biomarker should raise suspicion of ICI-associated myocarditis. Notably, complete heart block has been observed in 36% of affected patients and in up to 64% of fatal cases, underscoring the importance of regular ECG monitoring in patients receiving ICIs (30). LVEF abnormalities are observed in 18%–41% of patients (32). However, a normal LVEF does not necessarily indicate mild disease; notably, 38% of patients with normal LVEF present with fulminant myocarditis (33). Cardiac MRI is a crucial noninvasive modality for diagnosing myocarditis, typically revealing focal myocardial edema with late gadolinium enhancement (34). Endomyocardial biopsy (EMB), the diagnostic gold standard, typically reveals lymphocytic and CD68-positive macrophage infiltration with varying degrees of myocardial injury (35). However, due to its invasive nature and associated procedural risks, EMB is rarely performed in clinical practice (36–38).

In this case, the temporal sequence and evolution of cardiac findings are instrumental in diagnosing ICI-associated myocarditis. Prior to immunotherapy, ECG revealed normal sinus rhythm without conduction defects, and echocardiography showed segmental wall motion abnormalities (hypokinesia of the inferoposterior wall of the left ventricle) consistent with the patient's known coronary heart disease. Following ICI therapy, the patient developed a new right bundle branch block, a marked extension and worsening of wall motion abnormalities to akinesis of the inferoposterior and lateral walls, and a significant elevation in cardiac troponin. The concurrence of a new conduction defect, progressive myocardial dysfunction extending beyond the baseline territory of coronary heart disease, and a sharp rise in cardiac biomarkers helps distinguish ICI-associated myocardial injury from the natural progression of underlying chronic ischemic heart disease. This clear temporal relationship supports a causal role of the immune checkpoint inhibitor.

The most common clinical presentation of ICI-associated MG is ocular and includes ptosis, diplopia, blurred vision, ophthalmoparesis, and neck and limb muscle weakness. Dysphagia or dyspnea may occur in severe cases. A neostigmine test may aid in the diagnosis. Compared with primary MG, ICI-associated MG progresses more rapidly and often presents with more severe symptoms. Repetitive nerve stimulation studies may yield positive results in these patients (27). Autoantibody testing has significant clinical value in the diagnosis of MG. The most commonly associated antibody is against the acetylcholine receptor (AchR); however, the overall positivity rate is only approximately 66% in ICI-associated MG (23), which is markedly lower than the 85%–90% positivity rate observed in non-ICI-related MG patients (39). Therefore, a negative anti-AChR antibody test result cannot rule out a diagnosis of ICI-associated MG.

A potential mechanism for the co-occurrence of ICI-associated MG and myocarditis is as follows: Shared high-frequency T-cell receptor (TCR) sequences have been found in both the myocardial and tumor tissues of these patients. It is speculated that T lymphocytes activated by ICI therapy may not only target tumors but also recognize common antigens shared by skeletal muscle, cardiac muscle, and normal nervous tissue, thereby inducing the onset of both conditions (40, 41). Compared with anti-PD-L1 and anti-CTLA-4 agents, PD-1 inhibitors (including nivolumab, pembrolizumab, and cemiplimab) exhibit the strongest signal for inducing myocarditis, suggesting the highest associated risk. Mahmood et al. (42) collected 35 cases of ICI-associated myocarditis between 2013 and 2017. After random sampling and matching, they found that compared with other ICIs, nivolumab and pembrolizumab accounted for the highest proportion of myocarditis adverse events, at 0.6% and 1.3%, respectively. Moslehi et al. (43) identified 101 patients diagnosed with ICI-associated myocarditis in the WHO Global Individual Case Safety Report database, of whom 57% reported developing myocarditis following PD-1 inhibitor therapy. Therefore, in cancer patients initiating anti-PD-1 immunotherapy, symptoms such as ptosis, chest pain, respiratory muscle weakness, dyspnea, or voice changes—with or without elevated laboratory markers such as troponin, CK, or CK-MB—should raise clinical suspicion for the co-occurrence of ICI-associated MG and myocarditis (44).

Patients with MG often have concomitant thymic disorders, including thymoma. As a key lymphoid organ, the thymus possesses immunological properties that predispose individuals with thymoma to associated autoimmune diseases, including MG. Notably, patients with cortical-type thymomas (including types B1, B2, and B3) exhibit a higher incidence of autoimmune diseases (45, 46). A study by Okumura et al. (46) reported that 43.7% (31/71) of patients with cortical-type thymoma developed MG, with the frequency reaching 55.6% (20/36) among those with type B2 thymoma. Furthermore, a retrospective study by Gutzmer et al. (47) demonstrated that 42% (8/19) of melanoma patients with a pre-existing autoimmune disease history experienced recurrence of their prior autoimmune condition following PD-1 inhibitor therapy. Thus, a history of autoimmune disease may be a predisposing factor for subsequent immune-related adverse events. In the present case, the patient had no prior history of autoimmune disease or thymoma. The symptoms of MG emerged after the first dose of an anti-PD-1 monoclonal antibody, with no other new medications introduced within the preceding month. Based on the literature and the clinical timeline, the MG in this patient is considered to have been induced by the anti-PD-1 monoclonal antibody.

In terms of management, the onset of myocarditis-MG overlap syndrome typically necessitates permanent discontinuation of ICIs and the initiation of aggressive immunomodulatory therapy, such as high-dose glucocorticoid pulses, intravenous immunoglobulin (IVIG), and plasma exchange (48). Major guidelines emphasize early high-dose corticosteroid therapy for ICI-associated MG or myocarditis, though specific recommendations vary. According to the 2023 Chinese Society of Clinical Oncology (CSCO) guidelines, management should be tiered based on severity: for Grade 1 (asymptomatic, with only biomarker or ECG abnormalities), ICI should be paused and methylprednisolone may be administered if necessary (1–2 mg/kg/day for 3–5 days), followed by a slow taper; for Grade 2 (mild symptoms or symptoms with moderate activity, accompanied by biomarker/ECG changes), immediate methylprednisolone (1–4 mg/kg/day) is advised, with tapering over at least 4–6 weeks after cardiac function returns to baseline; for Grade 3 (marked symptoms at rest/minimal activity, biomarkers >ULN, and clear echocardiographic/ECG abnormalities), methylprednisolone pulse therapy (500–1000 mg/day for 3 days) should be initiated, then switched to 1 mg/kg/day and tapered slowly over at least 4–6 weeks (49). The 2022 European Society of Cardiology (ESC) guidelines recommend intravenous methylprednisolone sodium succinate (500–1,000 mg/day for 3–5 days) within 24 hours, continuing until symptoms resolve, left ventricular function and conduction recover, and cardiac troponin declines by >50% from peak (50). Regarding neurologic immune-related adverse events, the 2018 American Society of Clinical Oncology (ASCO) guidelines suggest that for Grade 2 or higher MG [corresponding to Myasthenia Gravis Foundation of America [MGFA] clinical class I [ocular symptoms and findings only] or class II [mild generalized weakness]], corticosteroids equivalent to methylprednisolone 1–4 mg/kg/day should be initiated based on symptoms. For ICI-associated myocarditis specifically, ASCO recommends starting with methylprednisolone 1–2 mg/kg/day, followed by a slow taper and conversion to oral therapy (51).

Other immunomodulatory agents such as rituximab may serve as second-line treatment options for patients refractory to initial therapies (52). In our case, the diagnoses of MG and myocarditis were both well-established. The overall presentation was mild without life-threatening adverse events. The patient's symptoms and biomarkers improved following the cessation of ICI therapy, administration of glucocorticoids, and supportive care. This approach of using corticosteroids as first-line therapy is consistent with guideline recommendations for irAEs. Given that high-dose steroids may paradoxically exacerbate MG and precipitate a myasthenic crisis (19), the initial dose was set at methylprednisolone sodium succinate 120 mg via intravenous infusion.

This case report highlights that even rare irAEs can co-occur as an overlapping syndrome during ICI therapy, necessitating a high index of clinical suspicion and a multidisciplinary approach for early diagnosis and prompt intervention. Early and appropriate administration of glucocorticoids, other immunosuppressants, and/or non-pharmacological measures is crucial for improving patient outcomes.

## Data Availability

The datasets presented in this article are not readily available because of ethical and privacy restrictions. Requests to access the datasets should be directed to the corresponding author.
