# Supplementary material for: Case Report and literature review: immune checkpoint inhibitor-associated myasthenia gravis and myocarditis
Source: Front Cardiovasc Med. 2026 Mar 19;13:1764567. doi: 10.3389/fcvm.2026.1764567 (PMC13043362; doi:10.3389/fcvm.2026.1764567)
Supplement: Supplementary file 1 [file Datasheet1.pdf]

CARE Checklist for Case Reports

(Completed for the case entitled: *Case Report and literature review: Immune checkpoint inhibitor-associated myasthenia gravis and myocarditis*)

Topic	Item	Checklist Item Description	Section Heading
Title	1	The diagnosis or intervention of primary focus followed by the words "case report"	Title
Key Words	2	2–5 key words that identify diagnoses or interventions in this case report, including "case report"	Keywords
Abstract (no references)	3a	Introduction: What is unique about this case and what does it add to the scientific literature?	Abstract
	3b	Main symptoms and/or important clinical findings	Abstract
	3c	The main diagnoses, therapeutic interventions, and outcomes	Abstract
	3d	Conclusion—What is the main "take-away" lesson(s) from this case?	Abstract
Introduction	4	One or two paragraphs summarizing why this case is unique (**may include references**)	Introduction
Patient Information	5a	De-identified patient specific information	Case description
	5b	Primary concerns and symptoms of the patient	Case description
	5c	Medical, family, and psychosocial history including relevant genetic information	Case description
	5d	Relevant past interventions with outcomes	Case description
Clinical Findings	6	Describe significant physical examination (PE) and important clinical findings	Case description
Timeline	7	Historical and current information from this episode of care organized as a timeline	Table 1
Diagnostic Assessment	8a	Diagnostic testing (such as PE, laboratory testing, imaging, surveys)	Case description
	8b	Diagnostic challenges (such as access to testing, financial, or cultural)	Case description

Topic	Item	Checklist Item Description	Section Heading
	8c	Diagnosis (including other diagnoses considered)	Diagnostic Approach
	8d	Prognosis (such as staging in oncology) where applicable	Case description/Literature review
Therapeutic Intervention	9a	Types of therapeutic intervention (such as pharmacologic, surgical, preventive, self-care)	Therapeutic Management
	9b	Administration of therapeutic intervention (such as dosage, strength, duration)	Therapeutic Management
	9c	Changes in therapeutic intervention (with rationale)	Therapeutic Management
Follow-up and Outcomes	10a	Clinician and patient-assessed outcomes (if available)	Treatment Response and Follow-up
	10b	Important follow-up diagnostic and other test results	Treatment Response and Follow-up/Table 2/Figure 1E-G
	10c	Intervention adherence and tolerability (How was this assessed?)	Treatment Response and Follow-up
	10d	Adverse and unanticipated events	No new treatment-related adverse events occurred
Discussion	11a	A scientific discussion of the strengths AND limitations associated with this case report	Discussion
	11b	Discussion of the relevant medical literature **with references**	Discussion
	11c	The scientific rationale for any conclusions (including assessment of possible causes)	Discussion

Topic	Item	Checklist Item Description	Section Heading
	11d	The primary “take-away” lessons of this case report (without references) in a one paragraph conclusion	Discussion
Patient Perspective	12	The patient should share their perspective in one to two paragraphs on the treatment(s) they received	Treatment Response and Follow-up
Informed Consent	13	Did the patient give informed consent? Please provide if requested	Ethics statement
